# PyRates—A Python framework for rate-based neural simulations

**DOI:** 10.1371/journal.pone.0225900

**Published:** 2019-12-16

**Authors:** Richard Gast, Daniel Rose, Christoph Salomon, Harald E. Möller, Nikolaus Weiskopf, Thomas R. Knösche

**Affiliations:** 1 MEG and Cortical Networks Group, Max Planck Institute for Human Cognitive and Brain Sciences, Leipzig, Saxony, Germany; 2 Nuclear Magnetic Resonance Group, Max Planck Institute for Human Cognitive and Brain Sciences, Leipzig, Saxony, Germany; 3 Neurophysics Department, Max Planck Institute for Human Cognitive and Brain Sciences, Leipzig, Saxony, Germany; 4 Institute for Biomedical Engineering and Informatics, TU Ilmenau, Ilmenau, Thuringia, Germany; SUNY Downstate MC, UNITED STATES

## Abstract

In neuroscience, computational modeling has become an important source of insight into brain states and dynamics. A basic requirement for computational modeling studies is the availability of efficient software for setting up models and performing numerical simulations. While many such tools exist for different families of neural models, there is a lack of tools allowing for both a generic model definition and efficiently parallelized simulations. In this work, we present PyRates, a Python framework that provides the means to build a large variety of rate-based neural models. PyRates provides intuitive access to and modification of all mathematical operators in a graph, thus allowing for a highly generic model definition. For computational efficiency and parallelization, the model is translated into a compute graph. Using the example of two different neural models belonging to the family of rate-based population models, we explain the mathematical formalism, software structure and user interfaces of PyRates. We show via numerical simulations that the behavior of the PyRates model implementations is consistent with the literature. Finally, we demonstrate the computational capacities and scalability of PyRates via a number of benchmark simulations of neural networks differing in size and connectivity.

## Introduction

In the last decades, computational neuroscience has become an integral part of neuroscientific research. A major factor in this development has been the difficulty in gaining mechanistic insights into neural processes and structures from recordings of brain activity, without additional computational models. This problem is strongly linked to the actual signals recorded with non-invasive brain imaging techniques such as magneto- and electroencephalography (MEG/EEG) or functional magnetic resonance imaging (fMRI). Even though the spatiotemporal resolution of these techniques has improved throughout the years, they are still limited with respect to the state variables of the brain they can detect. Spatial resolution in fMRI has been pushed to the sub-millimeter range [1, 2], whereas EEG and MEG offer a temporal resolution thought to be sufficient to capture all electrophysiological signaling processes in the brain [3]. On the EEG/MEG side, the measured signal is thought to arise mainly from the superposition of primary and secondary currents resulting from post-synaptic polarization of a large number of cells with similarly oriented dendrites [4]. Therefore, the activity of cell-types that do not show a clear orientation preference (like most inhibitory interneurons [5]) are difficult to detect, even though they might play a crucial role for the underlying neural dynamics. Further issues of EEG/MEG acquisitions are their limited sensitivity to sub-cortical signal sources and the inverse problem one faces when trying to locate the source of a signal within the brain [6]. On the other hand, fMRI measures hemodynamic signals of the brain related to local blood flow, blood volume, and blood oxygenation levels and thus delivers only an indirect, strongly blurred view of the dynamic state of the brain [7]. These limitations create the need for additional models and assumptions that link the recorded signals to the underlying neural activity. Computational models of neural dynamics (called neural models henceforth) are particularly important for interpreting neuroimaging data and understanding the neural mechanisms involved in their generation [8–10]. Such models have been developed for various spatial and temporal scales of the brain, ranging from highly detailed models of a single neuron to models that represent the combined activity of thousands of neurons. In any case, they provide observation and control over all state variables included in a given model, thus offering mechanistic insights into their dynamics.

Numerical simulations are the primary method used to investigate neural models beyond pure mathematical analyses and to link model variables with experimental data. Such numerical simulations can be highly computationally expensive and scale with the model size, simulation time, and temporal resolution of the simulation. Different software tools have been developed for neural modeling that offer various solutions to render numerical simulations more efficient (e.g. TVB [11], DCM [12], Nengo [13], NEST [14], ANNarchy [15], Brian [16], and NEURON [17]). Since the brain is a highly parallelized information processing system (i.e. all of its $\tilde{1}00$ billion cells can transform and propagate signals in parallel), most models of the brain have a high degree of structural parallelism as well. This means that they involve calculations that can be evaluated in parallel, such as the updating of the firing rate of each cell population inside a neural model. One obvious way of optimizing numerical simulations of neural models is to distribute these calculations on parallel hardware, such as the central and graphical processing units (CPUs and GPUs) of a computer. Neural simulation tools that implement such mechanisms include Nengo [13], ANNarchy [15], Brian [16], NEURON [18], and PCSIM [19], for example. Each of these tools has been built for neural models of certain families. For example, the setup and simulation of complex multi-compartment models of single spiking neurons is supported by NEURON, Nest, and Brian. Tools dedicated to networks of point neurons, on the other hand, include ANNarchy, Nengo, and PCSIM (though NEURON, Nest, and Brian support point neuron models as well). Finally, neural population models are the focus of TVB and DCM. For most of these tools, a pool of pre-implemented models of the given families are available that the user can choose from. However, often it is not possible to add new models or modeling mechanisms to this pool without considerable effort. This holds true especially if one wants to benefit from the parallelization and optimization features of the respective software. Exceptions are tools like ANNarchy and Brian that include code generation mechanisms. These allow the user to define the mathematical equations that certain parts of the model will be governed by and will automatically translate them into the same representations that the pre-implemented models follow. Unfortunately, the tools that provide such code generation mechanisms are limited with regards to the model parts that can be customized in such a way and concerning the families of neural models they can express. It should be mentioned that NEURON allows one to build custom, multi-compartment neuron models that can be used in network models without any impact on the parallelization mechanisms. This enables the setup of heterogeneous, multi-scale models of single cells without loss of parallelization efficiency via high-level interfaces to NEURON such as BioNet or NetPyNE [20, 21]. However, these mechanisms do not allow the modification of the underlying equations of the state variables in the model.

To summarize, we believe that the increasing number of computational models and numerical simulations in neuroscientific research necessitates the development of neural simulation tools that:

- follow a well-defined mathematical formalism in their model configurations,
- are flexible enough so that scientists can implement custom models that go beyond pre-implemented models in both the mathematical equations and network structure,
- are structured in such a way that models are easily understood, set up, and shared with other scientists,
- enable efficient numerical simulations on parallel computing hardware.

To address these needs, we present PyRates, an open-source Python framework for rate-based neural modeling (freely available at https://www.cbs.mpg.de/departments/neurophysics/software/pyrates and https://github.com/pyrates-neuroscience/PyRates). The basic aim behind PyRates is to provide a well-documented, thoroughly tested, and computationally powerful framework for neural modeling and simulations. In PyRates, both the model configuration and simulation can be performed with a few lines of code. Each model is represented by a graph of nodes and edges, with the former representing the model units (i.e. single cells, cell populations, …) and the latter the information transfer between them. Further, as we will explain in more detail below, the user has full control over the mathematical equations that define the nodes and edges. To enable an efficient parallelization, the underlying model equations are translated into a compute graph, specifying which parts of the equations have to be evaluated serially and which parts can be processed in parallel. Parallel hardware that PyRates can employ for this purpose includes central processing units (CPUs), graphical processing units (GPUs), and compute clusters with multiple machines. In principle this will allow the implementation of any kind of dynamic neural system that can be expressed as a graph. For the remainder of this article we will focus on a specific family of neural models, namely rate-based population models (hence the name PyRates).

The focus on population models is (i) in accordance with the expertise of the authors and (ii) serves the purpose of keeping the article concise. However, the emphasis of the paper lies on introducing the features and capacities of the framework, how to define a model in PyRates, and how to use the software to perform and analyze neural simulations. Therefore, we first introduce the mathematical syntax used for all of our models, followed by an explanation how single mathematical equations are structured in PyRates to form a neural network model. To this end, we provide a step-by-step example of how to configure and simulate a particular neural population model. We continue with a section dedicated to the evaluation of different numerical simulation scenarios. First, we validate the implementation of two exemplary neural population models in PyRates by replicating key behaviors of the models reported in their original publications. Second, we demonstrate the computational efficiency and scalability of PyRates via a number of benchmarks that constitute realistic numerical simulation scenarios. Finally, we discuss the strengths and limitations of PyRates for developing and simulating neural models.

## Neural population models

Investigating the human brain via EEG/MEG or fMRI means working with signals that are assumed to represent changes in the average activity of large cell populations. While these signals can be explained by detailed models of single cell processes, such models come with a state space of much higher dimensionality than the measured signals. Indeed, several approaches exist that employ this strategy to model the neural processes underlying macroscopic brain signals [22, 23] via tools such as the *Human Neocortical Neurosolver* or *LFPy* [24, 25]. As an alternative approach, neural mass models have widely been used to model the dynamics of the macroscopic brain signals of interest [26]. That is, they describe the average activity of large cell populations in the brain via a mean-field approach, rendering their investigation computationally much less expensive than single cell approaches [8, 27, 28]. As a downside, all information about the underlying single cell activity, except for the mean of their probability distribution is lost. Thus, their application is limited to neurodynamic questions addressing changes in those macroscopic state variables. Often, neural mass models express the state of each neural population by an average membrane potential and an average firing rate. The dynamics and transformations of these state variables can typically be formulated via three mathematical operators. The first two describe the input-output structure of a single population: While the rate-to-potential operator (RPO) transforms synaptic inputs into average membrane potential changes, the potential-to-rate operator (PRO) transforms the average membrane potential into an average firing rate output. Widely used forms for these operators are a convolution operation with an exponential kernel for the RPO (e.g. [29, 30, 32]) and a sigmoidal, instantaneous transformation for the PRO (e.g. [28, 33, 34]). The third operator is the coupling operator (CO) that transforms outgoing into incoming firing rates and is thus used to establish connections across populations. By describing the dynamics of large neural population networks via three basic transforms (RPO, PRO & CO), neural mass models combine computational feasibility with biophysical interpretability. Due to these desirable qualities, neural mass models have become an attractive method for studying neural dynamics on a meso- and macroscopic scale [8, 10, 26]. They have been established as one of the most popular methods for modeling EEG/MEG and fMRI measurements and have been able to account for various dynamic properties of experimentally observed neural activity [31, 32, 35–40].

**Fig 1:**
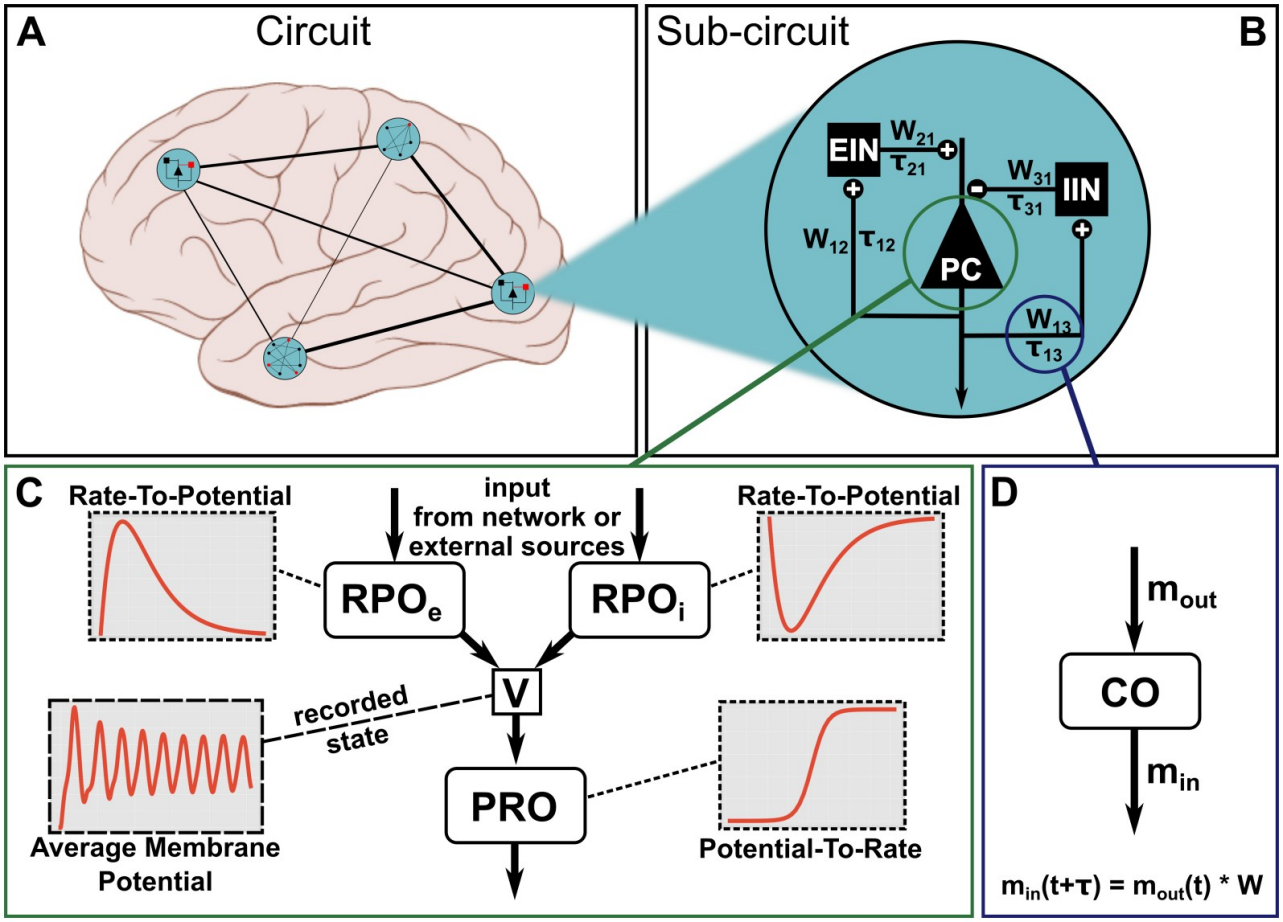
Model structure in PyRates. The largest organizational unit of a network model is the *Circuit*. Any circuit may also consist of multiple hierarchical layers of subcircuits. Panel (A) depicts an imaginary circuit that encompasses four subcircuits that represent one brain region each. One of these local circuits is a Jansen-Rit circuit (B), consisting of three neural populations (PC, EIN, IIN) and the connections between them. One node (C) may consist of multiple operators containing the mathematical equations. Here, two rate-to-potential operators (RPO) convolute incoming firing rates with an alpha kernel to produce post-synaptic potentials. These are summed into a combined membrane potential *V*. The potential-to-rate operator (PRO) transforms *V* into an outgoing firing rate *m*_*out*_ via a sigmoid function. Inset graphs give a qualitative representation of the operators and evolution of the membrane potential. Edges (lines in A and B) represent information transfer between nodes. As panel (D) shows, edges may also contain operators. By default, edges apply a multiplicative weighting constant and can optionally delay the information passage with respect to time. The equation shown in panel (D) depicts this default behavior.

A particular neural mass model that we will use repeatedly in later sections is the three-population circuit introduced by Jansen and Rit [29]. The Jansen-Rit circuit (JRC) was originally proposed as a mechanistic model of the EEG signal generated by the visual cortex [29, 41]. Historically, however, it has been used as a canonical model of cell population interactions in a cortical column [35, 36, 40]. Its basic structure can be seen in Fig 1B, which can be thought of as a single cortical column. The signal generated by this column is the result of dynamic interactions between a projection cell population of pyramidal cells (PC), an excitatory interneuron population (EIN) and an inhibitory interneuron population (IIN). For certain parametrizations, the JRC has been shown to be able to produce key features of a typical EEG signal, such as the waxing-and-waning alpha oscillations [29, 30, 42]. A detailed account of the model’s mathematical description will be given in the next section, where we will demonstrate how to implement models in PyRates, using the example of the JRC equations. We chose to employ the JRC as an exemplary population model in this article since it is an established model used in numerous publications that the reader can compare with our report.

Another neural population model that we will make use of in this paper is the one described by Montbrió and colleagues [43]. It has been mentioned as one of the next generation neural mass models that provide a more precise mean-field description than classic neural population models like the JRC [44]. The model proposed by Montbrió and colleagues represents a mathematically exact mean-field derivation of a network of globally coupled quadratic integrate-and-fire neurons [43]. It can thus represent every macroscopic state the single cell network may fall into. This distinguishes it from the JRC, since it has no such correspondence between a single-cell network and the population descriptions. Furthermore, the macroscopic states (average membrane potential and average firing rate) of the Montbrió model can be linked directly to the synchronicity of the underlying single-cell network, a property that benefits the investigation of EEG phenomena such as event-related (de-)synchronization. We chose this model as our second example case due to its novelty and its potential importance for future neural population studies. Within the domain of rate-based neural population models, we found these two models sufficiently distinct to demonstrate the ability of PyRates to implement different model structures.

## The framework

PyRates requires an installation of Python 3.6 or newer and can be installed via the package manager *pip*, simply by calling pip install pyrates from the command line. The core goal of PyRates is to let scientists focus on the model definition, *i.e*. working out the equation structure, while the software takes care of transforming them into computationally efficient network structures and numerical simulations thereof.

**Fig 2:**
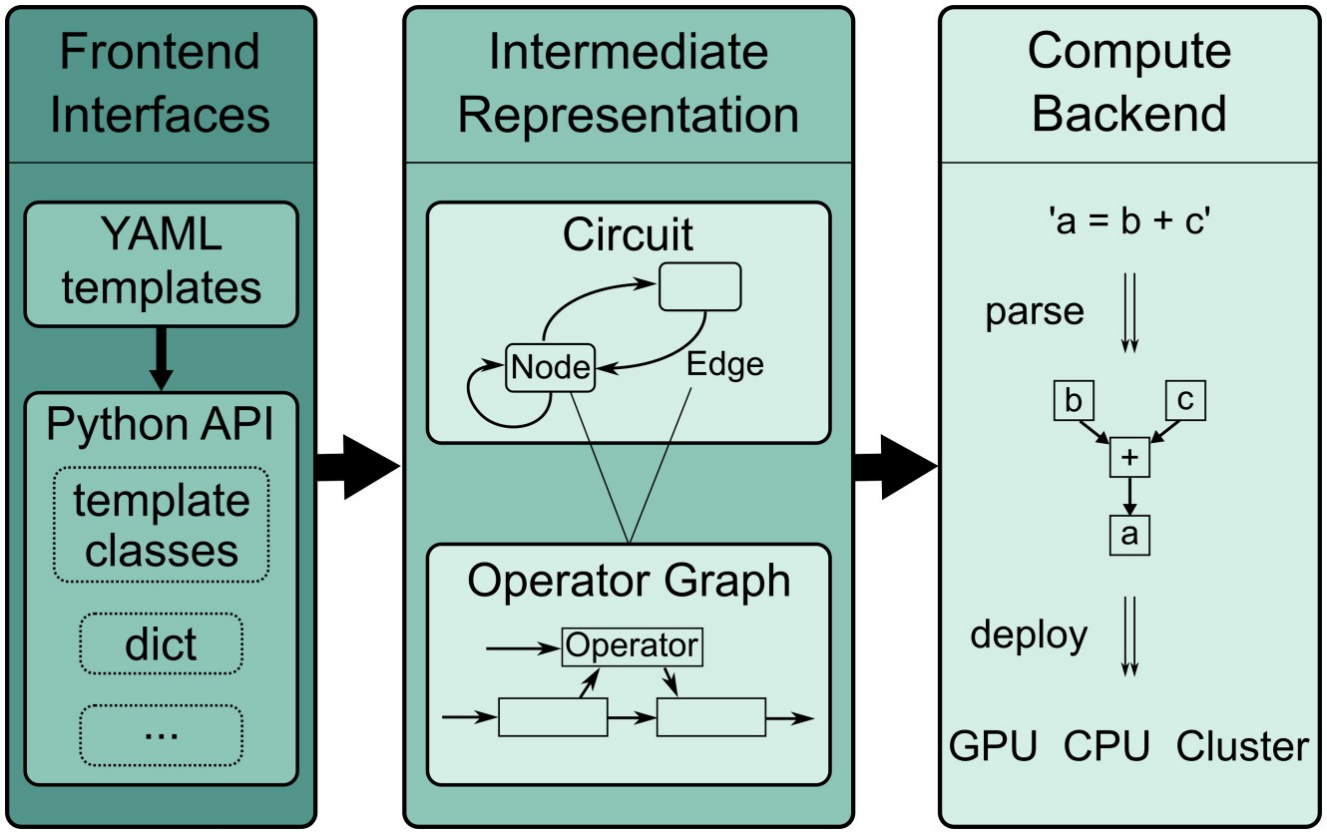
Schematic of software layers. PyRates is separated into frontend, intermediate representation (IR) and backend. The frontend features a set of interfaces to define network models. These are then translated into a standardized structure, called the IR. Simulations are realized via the backend, which transforms the high-level IR into lower-level representations for efficient computations. The frontend can easily be extended with new interfaces, while the backend can be swapped out to target a different computation framework.

This goal is reflected in the modular software design and user interface. Model configuration and simulation are realized as separate software layers as depicted in Fig 2. The *frontend* features multiple user interfaces for different levels of programming expertise and allows scientists to flexibly implement custom models. The models are then transformed into a graph-based *intermediate representation* that the *backend* interprets to perform efficient computations. We employ a custom mathematical syntax and domain specific model definition language. Both focus on readability and are much reduced in comparison to general-purpose languages. The following paragraphs explain the user interfaces and how to define models and run simulations. More details on implementation and installation can be found in the online documentation (see pyrates.readthedocs.io).

### Mathematical syntax

Neural network models are usually defined by a set of (differential) equations and corresponding parameters. In PyRates, researchers can define computational models in terms of algebraic equations and relations between different equations. The mathematical syntax strongly follows the conventions used in Python, though in some cases common alternatives are allowed as well. For example, the equation $a=\frac{5\cdot (b+c)}{d^{2}}$ can be written as a = 5 * (b + c) / d**2. Here, the power operator is a double asterisk ** as used in Python. However, the commonly used caret ^ symbol is implemented as a synonym. Parentheses, for example (b + c) indicate grouping. Arguments to a function are also grouped using parenthesis, e.g. exp(2) or sin(4 + 3).

Currently, PyRates does not include a full computer algebra system. By convention, the variable of interest is positioned on the left-hand-side of the equality sign and all other variables and operations on the right-hand-side. First-order differential equations are allowed as an exception: The expression d/dt * a is treated as a new variable and can thus be positioned as the variable of interest on the left-hand-side as in

$$\begin{array}{c} \text{d}/\text{dt}*\text{a}=\text{a}+\text{d} \end{array}$$ (1)

As a short-hand synonym, the expression a’ may be used as well (e.g. *a*′ = *a* + *d*). Higher order differential equations must be given as a set of coupled first-order differential equations. For example the equation

$$\begin{array}{c} \frac{d^{2}a}{dt^{2}}+\frac{da}{dt}+a=b+c \end{array}$$ (2)

can be reformulated as the following set of two coupled first-order differential equations:

$$\begin{array}{ll} \frac{da}{dt}=x & \Leftrightarrow \text{d}/\text{dt}*\text{a}=\text{x} \end{array}$$ (3)

$$\begin{array}{ll} \frac{dx}{dt}=b+c-x-a & \Leftrightarrow \text{d}/\text{dt}*\text{x}=\text{b}+\text{c}-\text{x}-\text{a} \end{array}$$ (4)

In simulations, this type of equation will be integrated for each time step of size *dt*. The following is an example for equations of a single neural mass in the classic Jansen-Rit model [41], which will be reused in later examples:

$$\begin{array}{ll} \text{RPO}: & \text{d}/\text{dt}*\text{V}\_\text{t}=\text{h}/\text{tau}*\text{r}\_\text{in}-1/\text{tau}**2*\text{V}-2*1/\text{tau}*\text{V\_t} \end{array}$$ (5)

d/dt*V=V_t (6)

$$\begin{array}{ll} \text{PRO}: & \text{r\_out}=\text{r\_max}/\left(1+\text{exp}\left(\text{s}*\left(\text{V\_thr}\;-\;\text{V}\right)\right)\right) \end{array}$$ (7)

Eq (7) represents the transformation of the population-average membrane potential *V* to an outgoing firing rate *r*_*out*_ via a sigmoidal transformation with slope *s*, maximum firing rate *r*_*max*_ and firing threshold *V*_*thr*_. This formulation contains a function call to the exponential function via exp(…). Using the pre-implemented sigmoid function, Eq (7) can be shortened to

$$\begin{array}{c} r\_\text{out}=r\_\text{max}*\text{sigmoid}\left(s*\left(V-V\_\text{thr}\right)\right) \end{array}$$ (8)

Multiple arguments to a function call are comma separated, e.g. in the sum along the first axis of matrix *A* which would be: sum(A, 0). Using comparison operators as function arguments, it is also possible to encode events, e.g. a spike, when the membrane potential *V* exceeds the threshold *V*_*thr*_:

$$\begin{array}{c} \text{spike}=\text{float}(V>V\_\text{thr}) \end{array}$$ (9)

The variable spike takes the decimal value 1.0 in case of a spike event and 0.0 otherwise.

The above examples assumed scalar variables, but vectors and higher-dimensional variables may also be used in PyRates. In particular, indexing is possible via square brackets […] and mostly follows the conventions of *numpy* [45], the *de facto* standard for numerics in Python. Supported indexing methods include single element indexing a[3], slicing [1:5], slicing along multiple axes separated by commas [0:5, 3:7], multi-element indexing a[[3], [4]], and slicing via Boolean masks a[a>5] for variable a of suitable dimensions. For more detailed explanations, please refer to the numpy documentation. A full list of supported mathematical symbols and pre-implemented functions can be found in the supporting information (S1 and S2 Tables).

### Components of a network model

In contrast to most other neural simulation frameworks, PyRates treats network models as network graphs rather than matrices. This works well for densely connected graphs, but gives the most computational benefit for sparse networks. Fig 1 gives an overview of the different components that make up a model. A network graph is called a *circuit* and is spanned by *nodes* and *edges*. For a neural population model, one node may correspond to one neural population with the edges encoding coupling between populations. In addition, circuits may be nested arbitrarily within other circuits. Small, self-contained network models can thus easily be reused in larger networks with a clear and intuitive hierarchy. Fig 1A illustrates this feature with a fictional large-scale circuit which comprises four brain areas and connections between them. Each area may consist of a single node or a more complex *sub-circuit*. Edges between areas are depicted as lines. Fig 1B zooms in on one brain area containing a three-node sub-circuit. This local model corresponds to the previously defined Jansen-Rit model [29, 41].

An individual network node consists of *operators*. One operator defines a scope, in which a set of equations and related variables are uniquely defined. It also acts as an isolated computational unit that transforms any number of input variables into one output. Whether an equation belongs to one operator or another decides the order in which equations are evaluated. Equations belonging to the same operator will be evaluated simultaneously, whereas equations in different operators can be evaluated in sequence. As an example, Fig 1C shows the operator structure of a pyramidal cell population in the Jansen-Rit model. There are two rate-to-potential operators (Eqs (5) and (6)), one for inhibitory synapses (RPO_i_) and one for excitatory synapses (RPO_e_). The two RPOs contain identical equations but different values assigned to the parameters. The subsequent potential-to-rate operator (PRO, Eq (7)) sums both synaptic contributions into one membrane potential that is transformed into an outgoing firing rate. In this configuration, the two synaptic contributions are evaluated independently, but possibly in parallel. The equation in the PRO on the other hand will only be evaluated after the synaptic RPOs. The exact order of operators is determined based on the respective input and output variables.

Apart from nodes, edges may also contain coupling operators. An example is shown in Fig 1D. Each edge propagates information from a *source* node to a *target* node. In between, one or more operators can transform the relevant variable, representing coupling dynamics between source and target nodes. This could represent an axon or bundle of axons that propagates firing rates between neural masses. Depending on distance, location or myelination, these axons may behave differently, which is encoded in operators. Note that edges can read any one variable from a source population and can thus be used to represent dramatically different coupling dynamics than those described above.

The described distinction between circuits, nodes, edges and operators is meant to provide an intuitive understanding of a model while giving the user many degrees of freedom in defining custom models.

### Model definition language

PyRates provides multiple interfaces to define a network model (see Fig 2). *Templates* are building blocks that can be reused at multiple scales. Complex heterogeneous networks will consist of many different templates whereas large homogeneous networks may reuse a few templates many times. For brevity, we will focus on the *YAML*-based template interface which is most suitable for users with little programming expertise. *YAML* is a data serialization standard using a syntax that is reduced to the absolute necessities and focuses on readability (version 1.2, [46]).

All examples in this section are based on the popular Jansen-Rit model [29]. Additionally, we will briefly discuss the implementation of the Montbrió model [43] for completeness. The Jansen-Rit model is a three-population neural mass model whose basic structure is illustrated in Fig 1. The model is formulated in two state-variables: Average membrane potential *V* and average firing rate *r*. Incoming presynaptic firing rates *r*_*in*_ are converted to post-synaptic potentials via the rate-to-potential operator (RPO). In the Jansen-Rit model, this is a second-order, linear, ordinary differential equation:

$$\begin{array}{c} \text{RPO}:\;{\left(\frac{d}{dt}+\frac{1}{\tau }\right)}^{2}V\left(t\right)=\frac{h}{\tau }r_{in}\left(t\right) \end{array}$$ (10)

with synaptic gain *h* and lumped time constant *τ*. The population-average membrane potential is then transformed into a mean outgoing firing rate *r*_*out*_ via the potential-to-rate operator (PRO)

$$\begin{array}{c} \text{PRO}:\;\;r_{out}=\frac{r_{max}}{1+e^{s(V_{thr}-V)}} \end{array}$$ (11)

which is an instantaneous logistic function with maximum firing rate *r*_*max*_, maximum slope *s*, and average firing threshold *V*_*thr*_. The equations above define a neural mass with a single synapse type. Multiple sets of these equations are coupled to form a model with three coupled neural populations. For the two interneuron populations, Eq (10) represents synaptic excitation. The pyramidal cell population uses this equation twice with two different parametrizations, representing synaptic excitation and inhibition, respectively. This model can be extended to include more populations or to model multiple cortical columns or areas that interact with each other. For such use-cases PyRates allows for the definition of templates that can be reused and adapted on-the-fly. The following defines a *YAML*-template for a rate-to-potential operator that contains Eq (10):

**JansenRitSynapse**: *# name of the template*

 **description**: … *# optional descriptive text*

 **base**: OperatorTemplate *# parent template or Python class to use*

 **equations**: *# unordered list of equations*

  - ’d/dt * V = V_t’

  - ’d/dt * V_t = h/tau * r_in − (1./tau)^2 * V − 2.*1./tau*V_t’

 **variables**:

  *# additional information to define variables in equations*

  **r_in**:

   **default**: input *# defines variable type*

  **V**:

   **default**: output

  **V_t**:

   **description**: integration variable *# optional*

   **default**: variable

  **tau**:

   **description**: Synaptic time constant

   **default**: constant

  **h**:

   **default**: constant

Similar to Python, *YAML* structures information using indentation to improve readability. The *base* attribute may either refer to the Python class that is used to load the template or a parent template. Using the *equations* attribute, an unsorted list of string-based equations should be provided. These equations will be evaluated simultaneously during simulations and need to follow the above defined mathematical syntax. The *variables* attribute gives additional information regarding the variables used within *equations*. The only mandatory attribute of variables is *default* which defines the variable type, data type and initial value. Additional attributes can be defined, e.g. a *description* may help users to understand the template itself or variables in the equations.

For the Jansen-Rit model, it is useful to define sub-templates for excitatory and inhibitory synapses. These share the same equations, but have different values for the constants *τ* and *h* which can be set in sub-templates, e.g. (values based on [41]):

**ExcitatorySynapse**:

 **base**: JansenRitSynapse *# parent template*

 **variables**:

  **h**:

   **default**: 3.25e−3

  **tau**:

   **default**: 10e−3

Above, the *JansenRitSynapse* template is reused as the *base* template and only the relevant variables are adapted. A single neural mass in the Jansen-Rit model may be implemented as a network node with one or more synapse operators and one operator that the transforms average membrane potential to the average firing rate (PRO, Eq (11)/Eq (7)):

**PyramidalCellPopulation**:

 **base**: NodeTemplate *# Python class for node templates*

 **operators**:

  - **ExcitatorySynapse** *# output: V*

  - **InhibitorySynapse** *# output: V*

  - **PotentialToRateOperator** *# input: V*

This node template represents the neural population of pyramidal projection cells as depicted in Fig 1C. PyRates internally orders operators based on their input and output variables. This way, complex operator hierarchies can be built without any additional syntax as long as input and output variable names are consistent across all operators. In this example, two synapse operators receive input from other neural masses (or external sources), transforming firing rates *r* into membrane potentials *V* (rate-to-potential operators, RPO). The synapse operators are independent and on the same hierarchical level. Equations in these two operators can thus be evaluated in parallel. Both synapse operators define the membrane potential *V* as output. The potential-to-rate (PRO) operator on the other hand, receives *V* as input. This is recognised as a dependency and the PRO will be evaluated after the synapse operators have been processed.

Note that cyclic operator dependencies are not allowed. If necessary, self-edges can be used to connect variables to each other within one node, to implement cyclic dependencies.

As described earlier, circuits are used in PyRates to represent one or more nodes and their connecting edges. The following circuit template represents the Jansen-Rit model as depicted in Fig 1B:

**JansenRitCircuit**:

 **base**: CircuitTemplate

 **nodes**: *# list nodes and label them*

  **EIN**: ExcitatoryInterneurons

  **IIN**: InhibitoryInterneurons

  **PC**: PyramidalCellPopulation

 **edges**: *# assign edges between nodes*

  *# − [<source>, <target>, <template_or_operators>, <values>]*

  - **[PC/PRO/r_out, IIN/RPO_e/r_in, null, {weight**: 33.75}]

  - **[PC/PRO/r_out, EIN/RPO_e/r_in, null, {weight**: 135.}]

  - **[EIN/PRO/r_out, PC/RPO_e/r_in, null, {weight**: 108.}]

  - **[IIN/PRO/r_out, PC/RPO_i/r_in, null, {weight**: 33.75}]

The *nodes* attribute specifies which node templates to use and assigns labels to them. These labels are used in *edges* to define source and target, respectively. Each edge is defined by a list (square brackets) of up to four elements: (1) source specifier, (2) target specifier, (3) template (containing operators), and (4) additional named values or attributes. The format for source and target is <node_label>/<operator>/<variable>, i.e. an edge establishes a link to a specific variable in a specific operator within a node. Multiple edges can thus interact with different variables on the same node. Note that for brevity the operators were abbreviated here in contrast to the definitions above. In addition to source and target, it is possible to also include operators inside an edge that allow additional transformations specific to the coupling between the source and target variables. These operators can be defined in a separate edge template that is referred to in the third list entry. In this particular example, the entry is left empty (“null”). The fourth list entry contains named attributes, which are saved on the edge. Two default attributes exist: weight scales the output variable of the edge before it is projected to the target and defaults to 1.0; delay determines whether the information passing through the edge is applied instantaneously (i.e. in the next simulation time step) or after a discrete delay (defined in seconds). By default, no delays are set. Additional attributes may be defined, e.g. to adapt values of operators inside the edge.

In the above example, all edges project the outgoing firing rate *r*_*out*_ from one node to the incoming firing rate *r*_*in*_ of a different node, rescaled by an edge-specific weight. Values of the latter are taken from the original paper by Jansen and Rit [29]. This example with the given values can be used to simulate alpha activity in EEG or MEG.

Jansen and Rit also investigated how more complex components of visual evoked potentials arise from the interaction of two circuits, one representing visual cortex and one prefrontal cortex [29]. In PyRates, circuits can be inserted into other circuits alongside nodes. A template for the two-circuit example from [29] could look like this:

**DoubleJRCircuit**:

 **base**: CircuitTemplate

 **circuits**: *# define sub−circuits and their labels*

  **JRC1**: JansenRitCircuit

  **JRC2**: JansenRitCircuit

 **edges**: *# assign edges between nodes in sub−circuits*

  - **[JRC1/PC/PRO/r_out, JRC2/PC/RPO_e/r_in, null, {weight**: 10.,

                        **delay**: 0.0}]

  - **[JRC2/PC/PRO/r_out, JRC1/PC/RPO_e/r_in, null, {weight**: 10.,

                        **delay**: 0.0}]

Circuits are added to the template in the same way as nodes, the only difference being the attribute name *circuits*. Edges are also defined similarly. Source and target keys start with the assigned sub-circuit label, followed by the label of the population within that circuit and so on. For heterogeneous or small networks it makes sense to build the entire circuit hierarchy with templates. For large-scale networks, PyRates also allows the loading of a connectivity matrix from which to build the network. This is realized via the Python interface. Assuming that a JRC template has been set up containing the 3 nodes (PC, EIN, IIN), the syntax for adding edges from a matrix is:

jrc = circuit_template.**apply**()

jrc.add_edges_from_matrix(source_var=’RPO/m_out’,

            target_var=’RPO_e_pc/m_in’,

            nodes=[’PC’, ’EIN’, ’IIN’],

            weight = C)

Here, *C* refers to a 3 x 3 matrix containing the connection strengths. It is also possible to define entire models (or even templates) using mere Python. Similar to YAML templates, templates defined in Python can also be adapted when they are referenced, to perform minor tweaks instead of defining multiple templates for small variations. For more information on alternative ways to set up a network and further examples, we refer the interested reader to the online documentation at pyrates.readthedocs.io.

### From model to simulation

All frontend interfaces translate a user-defined model into a set of Python objects that we call the *intermediate representation* (IR, middle layer in Fig 2). This paragraph will give more details on the IR and explain how a simulation can be started and evaluated based on the previously defined model. A model circuit is represented by the CircuitIR class, which builds a network graph representation of the model using the software package *networkx* [47]. The package is commonly used for graph-based data representation in Python and provides many interfaces to manipulate, analyze and visualize graphs. The CircuitIR contains additional convenience methods to plot a network graph or access and manipulate its content. The following lines of code load the JansenRitCircuit template that was defined above and transforms the template into a CircuitIR instance:

**from** pyrates.frontend **import** CircuitTemplate

# read YAML template and convert to Python object

template = CircuitTemplate.from_yaml(“path/to/file/JansenRitCircuit”)

# transform template object to intermediate representation

circuit_ir = template.**apply**()

The apply method also accepts additional arguments to change parameter values while applying the template.

Actual simulations take place in the compute backend (see Fig 2). Currently, the user can choose between two backend implementations. The default backend is based on *NumPy* and provides particularly fast simulations on single CPUs and, in combination with the Python distribution provided by Intel, on multiple CPUs. The alternative backend is based on *tensorflow 2.0* [48], which makes use of dataflow graphs to run parallel computations on CPUs and GPUs. For optimal parallelization of network representations, PyRates can summarize identical sets of (scalar) mathematical operations into more efficient vector operations. Automatic vectorization can be enabled via the vectorization keyword argument of the compile method:

net = circuit_ir.**compile**(vectorization = True, dt = 0.0001, solver=’euler’)

where vectorization = False indicates that the model should be processed as is, while vectorization = True reduces identical nodes to one vectorized node. dt refers to the (integration) time step in seconds used during simulations. By default, differential equations are integrated using an explicit Euler algorithm which is the most common algorithm used in stochastic network simulations. In addition, PyRates provides two alternative numerical solvers that can be chosen via the solver argument. They implement the midpoint method (solver=’midpoint’) and a 2/3 Runge-Kutta algorithm (solver=’rk23’). The unit of dt and the choice of a suitable value depends on time constants defined in the model. Here, we chose a value of 0.1*ms*, which is consistent with the numerical integration schemes reported in the literature (e.g. [40, 43]). A simulation can be executed by calling the run method, e.g.:

results, time = net.run(simulation_time = 10.0, *# in seconds*

          outputs={’V’: ‘PC/PRO/V’},

          sampling_step_size = 0.01) *# in seconds*

This example defines a total *simulation time* of 10 seconds and specifies that only the membrane voltage from *PC* (pyramidal cell) nodes should be observed. Note that variable histories will only be stored for variables defined as output. All other data is overwritten as soon as possible to save memory. Along this line, a sampling step-size can be defined that determines the distance in time between observation points of the output variable histories. Collected data is formatted as a DataFrame from the *pandas* package [49], a powerful data structure for serial data that comes with a lot of convenience methods, e.g. for plotting or statistics. To gain any meaningful results from this implementation of a JRC, it needs to be provided input in a biologically plausible range. External inputs can be included via *input* variables. To allow for external input being applied pre-synaptically to the excitatory synapse of the pyramidal cells, one would have to modify the JansenRitSynapse as follows:

**JansenRitSynapse_with_input**:

 **base**: JansenRitSynapse

 **equations**:

  **replace**: *# insert u by replacing m_in by a sum*

   **r_in**: (r_in + u)

 **variables**:

  **u**: *# adding the new additional variable u*

   **default**: input

We reused the previously defined JansenRitSynapse template and added the variable u as an input variable by replacing occurrences of r_in by (r_in + u) using string replacement. The previously defined equation

d/dt*V_t=h/tau*r_in−(1./tau)^2*V−2.*1./tau*V_t

thus turns into

d/dt*V_t=h/tau*(r_in+u)−(1./tau)^2*V−2.*1./tau*V_t

This modification enables the user to apply arbitrary input to the excitatory synapse of the pyramidal cells, using the inputs parameter of the run method:

results, time = net.run(simulation_time = 10.0,

          outputs={’V’: ‘PC/PRO/V’},

          inputs={’PC/RPO_e/u’: ext_input})

In this example, ext_input would be an array defining the input value for each simulation step. This subsumes a working implementation of a single Jansen-Rit model that can be used as a base unit to construct models of cortico-cortical networks. By using the above defined *YAML* templates, all simulations described in the next section that are based on Jansen-Rit models can be replicated.

### Implementing the Montbrió model

The neural mass model recently proposed by Montbrió and colleagues is a single-population model that is derived from all-to-all coupled quadratic integrate-and-fire (QIF) neurons [43]. It establishes a mathematically exact correspondence between macroscopic (population level) and microscopic (single cell level) states and equations. The model consists of two coupled differential equations that describe the dynamics of mean membrane potential *V* and mean firing rate *r*:

$$\begin{array}{c} \frac{dr}{dt}=\frac{\Delta }{\pi \tau^{2}}+\frac{2rV}{\tau } \end{array}$$ (12)

$$\begin{array}{c} \frac{dV}{dt}=\frac{1}{\tau }(V^{2}+\bar{\eta }+I\left(t\right))+Jr-\tau \pi^{2}r^{2} \end{array}$$ (13)

with intrinsic coupling *J* and input current *I*(*t*). Δ and $\bar{\eta }$ may be interpreted as the spread and mean of the distribution of excitability levels within the population. Note that the time constant *τ* was set to 1 and hence omitted in the derivation by Montbrió and colleagues [43]. The following operator template implements these equations in PyRates:

**MontbrioOperator**:

 **base**: OperatorTemplate

 **equations**:

  - “d/dt * r = delta/(PI * tau**2) + 2.*r*V/tau”

  - “d/dt * V = (V**2 + eta + inp) / tau + J*r − tau*(PI*r)**2”

 **variables**:

  …

Variable definitions are omitted in the above template for brevity. Since a single population in the Montbrió model is already capable of oscillations, a meaningful network can be set up with a single neural mass as follows:

**MontbrioPopulation**:

 **base**: NodeTemplate

 **operators**:

  - **MontbrioOperator**

**MontbrioNetwork**:

 **base**: CircuitTemplate

 **nodes**:

  **Pop1**: MontbrioPopulation

 **edges**:

This template can be used to replicate the simulation results presented in the next section that were obtained from the Montbrió model.

### Exploring model parameter spaces

When setting up computational models, it is often important to explore the relationship between model behavior and model parametrization. PyRates offers a simple but efficient mechanism to run many such simulations on parallel computation hardware. The function pyrates.utility.grid_search takes a single model template along with a specification of the parameter grid to sample sets of parameters from. It then constructs multiple model instances with differing parameters and adds them to the same circuit, but without edges between individual instances. All model instances can thus be computed efficiently in parallel on the same parallel hardware instead of executing them consecutively. How many instances can be simulated on a single piece of hardware depends on the memory capacities and number of parallel compute units. Additionally, PyRates provides an interface for deploying large parameter grid searches across multiple work stations. This allows the splitting of large parameter grids into smaller grids that can be run in parallel on multiple machines. For a tutorial on how to use those functionalities, we refer the interested reader to the jupyter notebooks that can be found at https://github.com/pyrates-neuroscience/PyRates/tree/master/documentation which contain various examples of parameter grid searches.

### Visualization and data analysis

PyRates features built-in functions for quick data analysis and visualization as well as native support for external libraries due to its commonly used data structures. On the one hand, network graphs are based on *networkx* Graph objects [47]. Hence, the entire toolset of networkx is natively supported, including an interface to the *graphviz* [50] library. Additionally, we provide functions for quick visualization of a network model within PyRates. On the other hand, simulation results are returned as a *pandas.DataFrame* which is a widely adopted structure for tabular data with powerful built-in analysis methods [49]. While this data structure already allows for an intuitive interface to the *seaborn* plotting library by itself, we also provide a number of visualization functions such as time-series plots, heat maps, and polar plots in PyRates. Most of those provide direct interfaces to plotting functions from *seaborn* and *MNE-Python*, the latter being an analysis toolbox for EEG and MEG data [51, 52].

## Results

The aim of this section is to (1) demonstrate that numerical simulations of models implemented in PyRates show the expected results and (2) analyze the computational capabilities and scalability of PyRates on a number of benchmarks. As explained previously, we chose the models proposed by Jansen and Rit and Montbrió and colleagues as exemplary models for these demonstrations. We will replicate the basic model dynamics under extrinsic input as reported in the original publications. To this end, we will compare the relationship between changes in the model parametrization and the model dynamics with the relationship reported in the literature. For this purpose, we will use the grid search functionality of PyRates, allowing evaluation of the model behavior for multiple parametrizations in parallel. Having validated the model implementations in PyRates, we will use the JRC as base model for a number of benchmark simulations. All simulations performed throughout this section use an explicit Euler integration scheme with a simulation step size of 0.1 ms. They have been run on a custom Linux machine with an NVidia Geforce Titan XP GPU with 12GB G-DDR5 graphic memory, a 3.5 GHz Intel Core i7 (4th generation) and 16 GB DDR3 working memory. Note that we provide Python scripts that can be used to replicate all of the simulation results reported below. They are available at https://github.com/pyrates-neuroscience/PyRates/tree/master/documentation.

### Validation of model implementations

#### Jansen-Rit circuit

**Fig 3:**
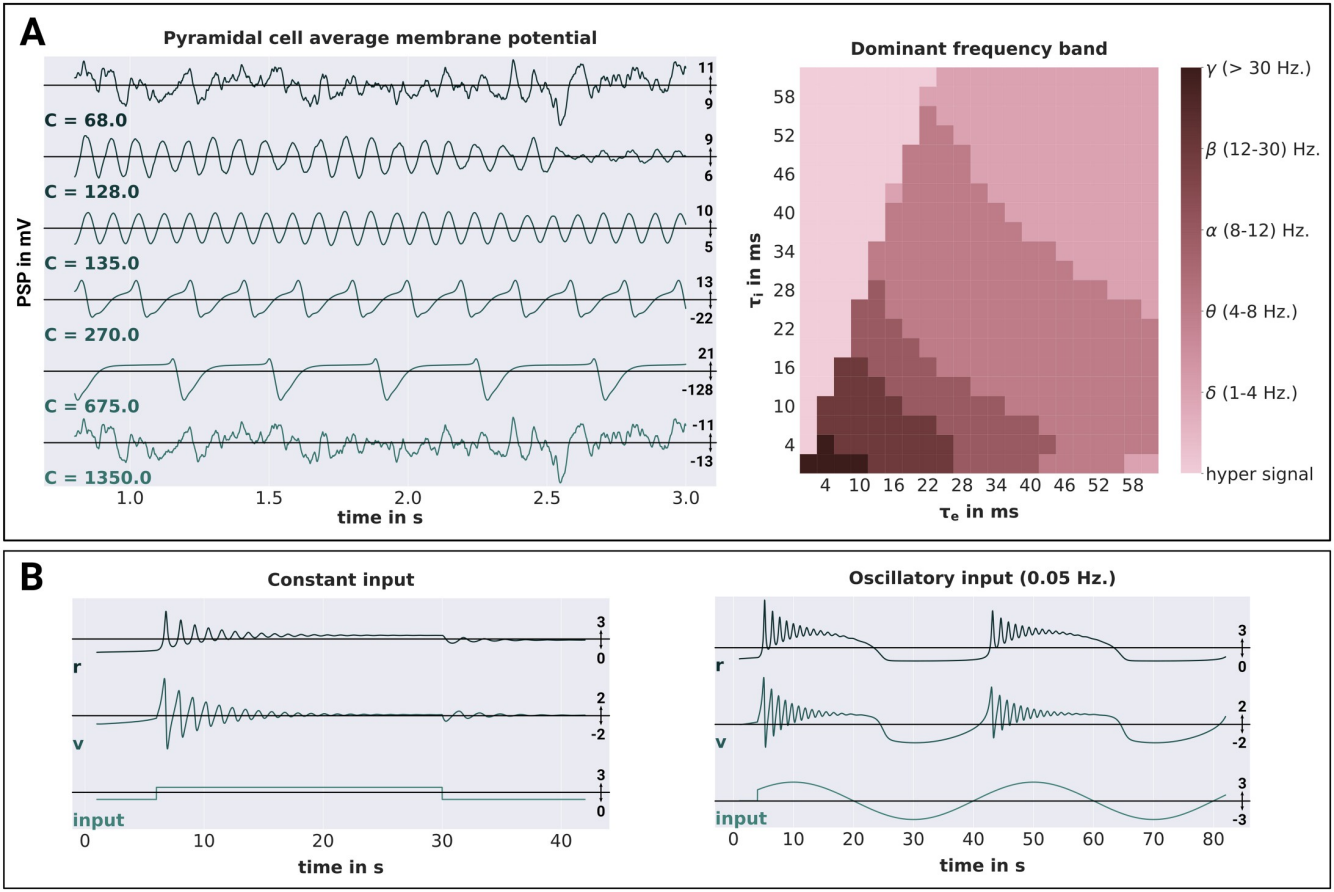
Jansen-Rit and Montbrió model validations. **A** Shows the simulation results obtained from a single Jansen-Rit model. On the left hand side, the average membrane potentials of the pyramidal cell population are depicted for different connectivity scalings C. On the right hand side, the dominant oscillation frequency of the pyramidal cell membrane potentials (evaluated over a simulation period of 60 seconds) is depicted for different synaptic time-scales *τ*_*e*_ and *τ*_*i*_. The frequencies are categorized into the following bands: *δ* (1-4 Hz), *θ* (4-8 Hz), *α* (8-12 Hz), *β* (12–30 Hz), *γ* (> 30 Hz) and h.s. (hyper signal) for signals not representative of any EEG component. **B** Shows the simulation results obtained from a single Montbrió model. The average membrane potentials *v*, average firing rates *r* and input currents are depicted for constant and oscillatory input on the left and right hand side, respectively. Time-dependent variables are reported in units of *τ*, which was set to *τ* = 1.0 in accordance with the simulations performed by Montbrió and colleagues. Following the definitions of Montbrió and colleagues, membrane potential and input are reported as unit-less variables.

The Jansen-Rit circuit has been shown to be able to produce a variety of steady-state responses [29, 30, 42]. In other words, the JRC has a number of bifurcation parameters that can lead to qualitative changes in the model’s state dynamics. In their original publication, Jansen and Rit delivered random synaptic input between 120 and 320 Hz to the projection cells while changing the scaling of the internal connectivities *C* [29] (reflected by the parameters *C*_*xy*_ in Fig 1B). As visualized in Fig 3 of [29], the model produced (noisy) sinusoidal oscillations in the alpha band for connectivity scalings *C* = 128 and *C* = 135, thus reflecting a major component of the EEG signal in primary visual cortex. For other scalings, it produced either random noise (*C* = 68 and *C* = 1350) or large-amplitude spiking behavior (*C* = 270 and *C* = 675). We chose to replicate this figure with our implementation of the JRC in PyRates. We simulated 2 s of JRC behavior for each internal connectivity scaling *C* ∈ {68, 128, 135, 270, 675, 1350}. All other model parameters were set according to the parameters chosen in [29]. The average membrane potential of the projection cell population (depicted as *PC* in Fig 1B) is depicted in the left panel of Fig 3A for each condition.

Results are in line with our expectations, showing random noise for both the highest and the lowest value of *C*, alpha oscillations for *C* = 128 and *C* = 135, and large-amplitude spiking behavior for the remaining conditions. Furthermore, the membrane potential amplitudes were in the same range as reported in [29] in each condition. Next to the connectivity scaling, the synaptic time scales *τ* of the JRC are further bifurcation parameters that have been shown to be useful to tune the model to represent different frequency bands of the brains’ EEG signal [30]. As demonstrated by David and Friston [30], varying these time scales between 1 and 60 ms leads to JRC dynamics that are representative of the delta, theta, alpha, beta and gamma frequency bands in the EEG. Due to its practical importance, we chose to replicate this parameter study as well. We systematically varied the excitatory and inhibitory synaptic timescales (*τ*_*e*_ and *τ*_*i*_) between 1 and 60 ms. For each condition, we adjusted the excitatory and inhibitory synaptic efficacies, such that the product *Hτ* was held constant. All other parameters were chosen as reported in [30] for the respective simulation. We then simulated the JRC behavior for 1 min and evaluated the maximum frequency of the power spectral density of the pyramidal cells membrane potential fluctuations. The results of this procedure are visualized in the right panel of Fig 3A. They are in accordance with the results reported in [30], showing response frequencies that range from the delta (1-4 Hz) to the gamma (> 30 Hz) range, as well as the hyper signal not representative of any EEG signal for too high ratios of $\frac{\tau_{i}}{\tau_{e}}$. Together, we are confident that our implementation of the JRC in PyRates accurately resembles the originally proposed model within the investigated dynamical regimes. Note, however, that faster synaptic time-constants or extrinsic input fluctuations should be handled carefully. For such cases, we recommend either reducing the above reported integration step size or choosing a more elaborate numerical solver (midpoint or Runge-Kutta 2/3) in order to avoid numerical instabilities.

#### Montbrió model

Even though the Montbrió model is only a single-population model, it has been shown to have a rich dynamic profile with bi-stable and even chaotic regimes [43, 53]. To investigate the response of the model to non-stationary inputs, Montbrió and colleagues initialized the model in a bi-stable dynamic regime and applied (1) constant and (2) sinusoidal extrinsic forcing within a short time-window. In the constant forcing condition they were able to show that the two different stable dynamic regimes of the model (stable focus and stable fixed point) could be switched between via a simple, transient step-function input. In the oscillatory forcing condition, on the other hand, they demonstrated that smooth changes in the extrinsic input was also able to cause the same state transitions in the model. This behavior can be observed in Fig 2 in [43] and we chose to replicate it with our implementation of the Montbrió model in PyRates. With all model parameters set to the values reported in [43] for this experiment, we simulated the model’s behavior for the constant and periodic forcing conditions. For both conditions, the external forcing strength was chosen as *I* = 30, while the frequency of the oscillatory forcing was chosen as $\omega =\frac{\pi }{20}$. Note that in accordance with the model definition of Montbrió and colleagues, time-dependent variables are reported in units of *τ* (which was set to *τ* = 1), while all other variables such as *v* and *I* are unit-less [43]. As shown in Fig 3B, we were able to replicate the above described model behavior. Constant forcing led to damped oscillatory responses of different frequency and amplitude at both onset and offset of the stimulus, whereas oscillatory forcing led to damped oscillatory responses around the peaks of the sinusoidal stimulus. Again, we take this as strong evidence for the correct representation of the Montbrió model by PyRates.

### Benchmarks

Neural simulation studies can differ substantially in the size and structure of the networks they investigate, leading to different computational loads. In PyRates, a number of backends and parallelization strategies are available for numerical simulations and their optimal choice may depend on the network architecture. In this paragraph, we describe how simulation durations in PyRates scale as a function of network size and connectivity and how this scaling behavior differs between different backends and parallelization types. For this purpose, we considered parallelization on a single machine vs. parallelized computations on multiple machines and simulations using the NumPy backend (CPU-based, version 1.17.2) vs. simulations using the tensorflow backend (supporting GPU parallelization, version 2.0.0-rc0).

**Fig 4:**
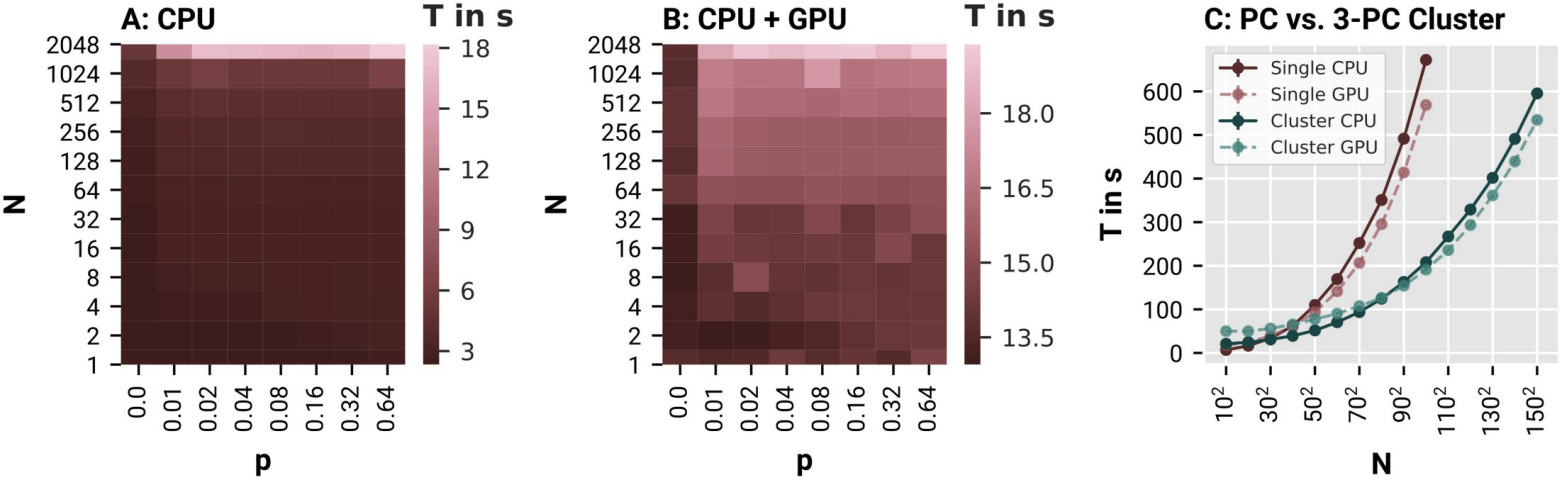
PyRates benchmarks. Benchmark results for 1 s simulations run in PyRates with a simulation step-size of 0.1 ms. **A** and **B** Show average simulation durations over 10 independent simulations for networks with different numbers of Jansen-Rit circuits (N) and differently dense coupling between the JRCs (p), performed on the NumPy (CPU) and tensorflow (CPU+GPU) backend, respectively. **C** Shows the average simulation durations for parameter sweeps over N different parametrizations of a network of 2 bidirectionally, delay-coupled Jansen-Rit circuits. Averages were again calculated over 10 independent runs of each parameter sweep.

In a first benchmark, we simulated the behavior of different JRC networks using either the NumPy or the tensorflow backend. Each network consisted of *N* ∈ {2^0^, 2^1^, 2^2^, …, 2^11^} randomly coupled JRCs with a coupling density of *p* ∈ {0.0, 0.25, 0.5, 0.75, 1.00}. Here, the latter refers to the relative number of pairwise connections between all pairs of JRCs that were established. Each JRC was parametrized such that it expressed waxing-and-waning alpha oscillations (*C* = 135.0; for all other parameters see [29]). The behavior of these networks was evaluated for a total of 1 s, leading to an overall number of 10^4^ simulation steps to be performed in each condition (given a step-size of 0.1 ms). To make the benchmark comparable to realistic simulation scenarios, we applied extrinsic input to each JRC and tracked the average membrane potential of every JRC’s projection cell population with a time resolution of 1 ms as output. Thus, the number of input and output operations also scaled with the network size. We assessed the time in seconds needed by PyRates to execute the run method of its backend in each condition, thus excluding the model initiation time. This was done via the Python internal package *time*. To account for random fluctuations due to background processes, we chose to report average simulation durations over *N*_*R*_ = 10 repetitions of each condition. To provide an estimate of these fluctuations, we calculated the average variation in the simulation duration *d* over conditions as $\sigma \left(d\right)=\frac{1}{N_{c}}\sum c\frac{\max\left(d_{c}\right)-\min\left(d_{c}\right)}{\langle d_{c}\rangle }$, with *c* being the condition index and 〈*d*〉 representing the expectation of *d*. We found average variations of *σ*(*d*) = 0.42*s* and *σ*(*d*) = 1.55*s* for the NumPy and tensorflow backend, respectively, which reflects the slightly stronger noise in the simulation duration we found for the tensorflow backend. The average simulation durations over conditions are visualized in Fig 4A and 4B for the NumPy and tensorflow backend, respectively. The average run times of the NumPy and tensorflow backend ranged between 2.5 and 18.1 seconds, and 13.2 and 20.3 seconds, respectively. Thus, the NumPy backend (running merely on the CPU) outperformed the tensorflow backend (running on CPU and GPU) on all considered network configurations. However, on large and densely connected networks, the tensorflow and NumPy backend expressed nearly the same simulation duration. This reflects the stronger parallelization capacities of the tensorflow backend, which is visible in its weaker scaling of the simulation duration with network size and coupling density. We expect this trend to lead to an advantage of the tensorflow backend for even larger networks. However, simulations of larger network sizes exceeded the working memory capacities of the machine we ran our benchmarks on. Together, these results demonstrate the effectiveness of PyRates’ backends in parallelizing network computations on CPUs and GPUs. While the NumPy backend showed the shortest run times for this benchmark, the tensorflow backend expressed less scaling behavior with the problem size. Thus, the latter might be superior in large-scale neural model simulations performed on a machine with better hardware configurations.

In a second benchmark, we examined the simulation time scaling in parameter sweeps performed via the grid search functionalities of PyRates on a single machine and on a cluster of 3 machines. The hardware specifications of each of those 3 machines were comparable to the ones reported in the beginning of this section. As an exemplary parameter sweep, we explored a parameter set which is prototypically investigated within the fields of connectomics and coupled oscillators, i.e. the connectivity scaling and propagation delay. To this end, we set up a network of 2 JRCs, with bidirectional coupling between their pyramidal cell populations. The bidirectional coupling was parametrized via a homogeneous coupling strength *κ* and a homogeneous propagation delay *τ* (in seconds). In each benchmark condition a parameter sweep was performed across all combinations of *κ* and *τ*. Thereby, the parameters were always varied within the ranges of *κ* ∈ [0.0, 200.0] and *τ* ∈ [0.0, 0.01], and only the number of steps between the limits of those ranges was varied across benchmark conditions. For example, a benchmark condition with 10 steps, would translate into a parameter sweep across all combinations of 10 different values of *κ* and *τ* and would hence result in *N* = 100 differently parametrized versions of the 2 coupled JRCs. All other parameters of the JRCs were the same as in the first benchmark. In each benchmark condition, 10 numerical simulation were performed for every network parametrization with a simulation time of *T* = 1*s*. Their average duration in dependence of *N* is visualized in Fig 4C for simulations performed on a single machine and on a 3-machine cluster, either using the NumPy or the tensorflow backend. Note that we also plotted the standard deviations across the 10 repetitions in each condition as error bars. However, those deviations were too small to be visible in Fig 4C. Also, these durations were in general larger than the ones reported in the first benchmark, because they include both the time to build the network and the time to perform the actual simulation. Since the network building process is not yet parallelized in PyRates, its duration shows stronger scaling behavior with the network size than the mere simulation times. As can be seen, the single machine outperformed the cluster for *N* < 900. Again, this can be explained by the overhead generated by the distribution of parameter chunks across the different machines and the collection of results from those machines after they finished their simulations. However, with increasing *N*, the benefit of parallelized simulations on multiple machines started to outweigh those costs, until reaching a maximum speed-up at *N* = 10000, where the 3-machine cluster was approximately 3 times faster than the single machine. This demonstrates that the maximal speed-up of parameter sweeps performed on compute clusters directly scales with the size of the cluster, which is a beneficial property for investigations of high-dimensional parameter spaces. In addition, Fig 4C shows that the speed-ups that resulted from different choices of backends were relatively small in comparison to the speed-ups achieved by running a parameter sweep on a single machine or on a cluster. This reflects the strong influence of the time it takes PyRates to build the network on the overall simulation duration *T*. Since these network building times do not differ between backends, we found a relatively small difference between NumPy and tensorflow backends in those parameter sweeps. Nonetheless, the tensorflow backend eventually outperformed the NumPy backend on large parameter sweeps (*N* ≥ 2500).

## Discussion

In this work we have presented PyRates, a novel Python framework for designing neural models and performing numerical simulations of their dynamic behavior. We introduced the frontend, including its user interfaces, structure, and mathematical syntax, and demonstrated how to build neural models, run numerical simulations, and perform parameter sweeps in PyRates. For validation purposes, we implemented the neural population models proposed by Jansen and Rit [29] and Montbrió and colleagues [43] and successfully replicated their key dynamic features. These results strongly suggest that both the model configurations produced by our frontend and their translation into compute graphs by our backend are accurate. Additionally, we tested the computational power of our backend on a number of different benchmarks. Those benchmarks consisted of simulations of JRC networks that differed in the number of their nodes and edges. We demonstrated that the CPU-based NumPy backend is most efficient for simulations of networks with up to a few thousand nodes, whereas the tensorflow backend (which can make use of GPUs) simulation durations showed the best scaling behavior with the problem size. The latter suggests an advantage of the tensorflow backend over the NumPy backend on large-scale neural network simulations with more than 10000 nodes. Indeed, we found the tensorflow backend to be more efficient on parameter sweeps over *N* ≥ 2500 parametrizations. Furthermore, we have shown how model parameter sweeps can benefit from parallelization on multiple machines.

From these results, we conclude that PyRates is a powerful simulation framework that enables highly efficient neural network simulations. The main questions we will address in the following discussion are (1) why is PyRates a valuable addition to established neural simulation software, and (2) in which cases can researchers benefit from using it.

### PyRates in the context of existing neural simulation frameworks

Within the domain of neural simulation frameworks, PyRates belongs to the family of graph-based neural simulators. In both its frontend and backend, it represents a neural model as a network of nodes connected by edges. PyRates makes no inherent assumptions concerning the spatial scale of nodes and edges in its networks, thus rendering it feasible for neural networks of any type. Additionally, PyRates allows for merging and hierarchical organization of neural networks by building graphs from sub-graphs. Hence, our tool can also be used to build multi-scale models, *e.g*. a macroscopic network of connected neural populations, with some populations of interest being represented by sub-networks of single neurons.

This being said, PyRates has only been systematically tested on rate-based population models. These differ qualitatively from spiking neuron models in terms of output variable, which is continuous for rate-based models but discrete for spiking neuron models. While it is in principle possible to implement such discrete spiking mechanisms, the compute engine is not optimized for it, since it projects output variables at each time-step to their targets in the network. This means that the projection operation will be performed regardless of whether a spike is produced or not, leading to considerable increases in computation time for large, densely connected, single cell networks. Hence, when dealing with neuroscientific questions that implicate the use of spiking neuron models, we currently recommend to use simulation tools such as Nengo [13], NEST [14], ANNarchy [15], Brian [16], NEURON [17], BioNet [20] or NetPyNE [21]. Such questions may involve problems where specific spike-timings have a non-negligible influence, where dendritic tree architectures are important or, more generally, where the variable of interest loses its meaning when averaged over time or over many neurons.

Of course, all of the above listed tools can be applied in other scenarios as well, even for macroscopic neural network simulations. However, if the variable of interest in a given model can be expressed as an average over many cells and single cell dynamics can be neglected, mean-field approaches such as the neural population models used throughout this article will be considerably faster and thus allow for the investigation of larger networks and parameter spaces. In general, most frameworks that feature generic code generation should allow the implementation of such models. From the above mentioned tools, Brian and ANNarchy belong to that category. Brian is strictly aimed at spike-based simulations and thus not optimized for continuous output variables like firing rates, whereas ANNarchy provides features for spike- and rate-based neural simulations. Nonetheless, it is designed for single-cell network simulations, so most of the templates it provides for neurons or populations are not necessarily applicable to mean-field models. Other simulation frameworks that provide explicit mean-field modeling mechanisms include TVB [54], DCM [12], DiPDE [55] and MIIND [56]. Among these, the latter two focus strongly on so-called population density techniques, which can describe the full voltage probability distribution of a population of neurons, instead of merely the mean. Both DiPDE and MIIND focus on the leaky integrate-and-fire neuron as the underlying model to derive the voltage probability distribution from. The advantage of this technique is the more direct and precise relationship between the single cell activity and the population level as compared to mean-field approaches. However, this advantage is payed for by higher computational demands, since a discretized probability distribution is computed at each simulation step instead of a mere point-estimate (i.e. the mean). TVB and DCM, on the other hand, focus on the same mathematical group of neurodynamic models as currently implemented in PyRates, i.e. neural population models. The focus of TVB lies in the simulation of large-scale brain networks via established, preferably homogeneous, local population models. DCM is explicitly designed to infer parameters of a fixed set of pre-implemented models based on a given measure of brain activity. While being the optimal choice for their respective use-cases, both tools lack functionalities that help when implementing custom models.

We consider the core strengths of PyRates to be its highly generic model definition (comparable to a pure code generation approach) and its two graph-based backends. The former distinguishes PyRates from other simulation frameworks, since it allows the customization of every part of a neural network, as long as a network structure with nodes and edges defined by mathematical operators is maintained. Every single computation that is performed in a PyRates simulation, and every variable that it uses, is defined in the frontend and can be accessed and edited by the user. This allows, for example, the addition of custom synapse types, plasticity mechanisms, complex somatic integration mechanisms, or even axonal cable properties. In addition, edges can access and connect all variables existing pre- or post-node, thus enabling the implementation of projections or plasticity mechanisms that depend on population variables other than firing rates. This generic approach makes PyRates particularly valuable for neuroscientists interested in developing novel neural models or extending existing ones.

A notion of caution should be added here. The degrees of freedom we provide for setting up models and simulations in PyRates imply that we do not provide safeguards for questionable model definitions. Except for their syntactical correctness, model equations and their hierarchical relationships will not be questioned further by PyRates. Also, inputs and outputs to the model will be added exactly as defined by the user. In other words, while PyRates does provide a considerable number of convenience functions to quickly set up and simulate large neural networks, it still requires users to be aware of potential numerical issues they could run into, if the model or simulation would not be set up correctly. Typical pitfalls include numerical overflows if variables become to large or small for the chosen data type, simulation step sizes that were chosen too large for the internal timescales of a given model, and random variables that are sampled at each simulation step without taking into account the dependency between sampling frequency and simulation step size. We tested numerical solvers providing adaptive time steps as an alternative to our fixed step size solvers to handle the problem of choosing an appropriate integration step size. However, we found those algorithms to be unsuited for network simulations in PyRates, since handling asynchronicity between network nodes created significant computational overhead.

Regarding PyRates’ second core strength, its backends, we have demonstrated its computational power in various scenarios. It provides optimized representations of large neural networks for simulations on CPUs and GPUs. Parallel execution of network simulations are particularly efficient when its nodes and edges are similar in their mathematical operators, since those similarities are exploited by the automatic vectorization mechanisms of PyRates. In turn, this means that the effectiveness of the parallelization scales negatively with the relative amount of heterogeneity or sequentiality of the network. Networks that consist of highly diverse neural units governed by many, hierarchically dependent operators will show considerably longer simulation durations than networks with very similar elements and a flat operator hierarchy. Thus, PyRates is particularly suited for simulating large, homogeneous networks or conducting parameter studies on small- to medium sized networks. For the latter, PyRates scales particularly well, since the size of the parameter sweep that can be computed in parallel grows with the size of the compute cluster among which our cluster distribution mechanism can distribute the different parametrizations.

### Integrating PyRates into neuroscientific work-flows

Neural population models such as the Jansen-Rit model [29] were originally conceived to understand or predict physical measures of brain activity such as LFPs, EEG/MEG or BOLD-fMRI. Modern neuroscientific workflows, however, go beyond forward simulations of brain activity. For example, The Virtual Brain [54] allows the use of structural (including diffusion-weighted) MRI scans to specify 3-dimensional structure and connectivity of a network design. Dynamic Causal Modeling [12] on the other hand can make use of measured brain activity to infer model parameters (e.g. connectivity constants) that best fit the given data. Both approaches have in common, that brain network models are adapted to individual subjects based on measured data.

PyRates integrates well with this concept for two reasons. (1) It is designed to provide an easy-to-use interface to construct and adapt network models with more flexibility than comparable tools. (2) Due to its modular software structure, PyRates can easily be extended to interface with existing tools. While the intermediate representation serves as a standard interface, the front- and backends can be exchanged to integrate with other software. For example, PyRates could be extended with a frontend that makes use of structural MRI data via tools provided by TVB. At the same time, the current backend could be extended to generate region-specific models compatible with TVB’s node model interface.

Currently, PyRates already provides a number of useful interfaces to tools that can be used for setting up models, subsequent analyses of simulated timeseries or model optimization. Two of those interfaces come with the graph representations PyRates uses for networks. As mentioned before, every PyRates network can either be translated into a NumPy- or tensorflow-based compute graph. This enables the usage of every NumPy or tensorflow function that could come in handy for setting up a model in PyRates, be it mathematical functions like *sine* or *max*, variable manipulation methods like *reshape* or *squeeze* or higher-level functions like error measurements or learning-rate decays. For the future, we also plan to provide interfaces to *tensorflow’s* model training features, which would allow to optimize parameters of neural models via gradient-descent based algorithms [48]. As an experimental feature, model parameter optimization is already possible via genetic algorithms, for which an interface is provided in the utility module of PyRates. They allow the definition of an arbitrary objective function for a given model and optimization of that function via subsequent model parameter updates employing mechanisms such as parameter re-combinations and mutations [57]. As with parameter sweeps, these algorithms can be executed either on a single or on multiple machines.

Since the intermediate representation fully builds on *networkx* graphs, the networkx API can be used to create, modify, analyze or visualize models. This includes interoperability with explicit graph visualization tools like Graphviz [49] or Cytoscape [58] that contain more elaborate features for visualizing complex biological networks. For the processing, analysis and visualization of simulation results, we provide a number of tools that mostly wrap *MNE-Python* [51, 52] and *seaborn* [59] functions. For extended use of *MNE-Python*, we also provide a wrapper that allows the translation of every output of a PyRates simulation into an *MNE-Python* object. This is particularly useful for forward simulations of EEG/MEG data, since *MNE-Python* comes with an extensive range of methods for the processing, analysis and visualization of such data. Finally, PyRates can also be used in combination with *pygpc*, a generalized polynomial chaos (GPC) toolbox for uncertainty quantification and sensitivity analysis publicly available under https://github.com/konstantinweise/pygpc. Via this interface it is possible to define a model plus a set of model parameters, including their respective uncertainties, and estimate how sensitive the model behavior is to changes in these parameters. It is important to note however, that the GPC cannot replace a proper bifurcation analysis and should currently only be used for parameter ranges where no bifurcations or multi-stabilities occur.

In summary, PyRates is readily integrated into complex neuroscientific workflows as a tool for bottom-up neural simulations. It provides interfaces to other Python tools that have been specifically designed to manage other parts of such workflows (e.g. data processing or visualization). More interfaces can easily be implemented due to the modular structure of the framework. This is further aided by the widely used data structures PyRates is built upon, like YAML-based configuration files, networkx graphs or pandas DataFrames. PyRates can thus be included as one independent component of larger neuroscientific workflows that can handle the definition, setup, numerical simulation and optimization of neural models.

## Supporting information

S1 Table Overview of mathematical syntax. (PDF)

S2 Table Overview of preimplemented mathematical functions. (PDF)
