# Supplementary material for: PyRates—A Python framework for rate-based neural simulations
Source: PLoS One. 2019 Dec 16;14(12):e0225900. doi: 10.1371/journal.pone.0225900 (PMC6913930; doi:10.1371/journal.pone.0225900)
Supplement: S1 Table — (PDF) [file pone.0225900.s001.pdf]

Supporting information

The mathematical syntax in PyRates is based on conventions used in the Python community. Table S1 summarizes the most common operations. Variable and function names consist of latin letters, underscores and numbers but must start with a letter or underscore. Indexing of higher-dimensional variables follows the numpy convention [40]. Table S2 shows preimplemented mathematical functions that are exposed in the mathematical syntax. Note that tensorflow may support more functions than PyRates exposes to the user. It is, however, straightforward to extend the list of exposed functions as needed. For a complete list of functions supported by tensorflow, please refer to the tensorflow API documentation [20].

Table S1. Overview of mathematical syntax.

Syntax	Description	Syntax	Description
**basic operations**		**reserved constants**	
a+b	addition	E, e	Euler‘s number
a-b	subtraction	PI	pi (π, Archimedes‘ constant)
a*b	multiplication	Parentheses	
a/b	division	(. . .)	prioritize operation, also function calls
a%b	modulo	a[. . .]	indexing
-a	negation	**indexing (following numpy standard)**	
a**b, a^b	exponent	a[b]	access element b of array a
**matrix operations**		a[b:c]	slice: access elements from b to c, excluding c. b or c may be left out.
a@b	matrix multiplication		
a.T	matrix transpose		
a.I	matrix inverse	a[b,c]	access element on index a_{b,c}
**relations**		a[[b], [c]]	multi-element indexing
a>b	greater	a[a>b]	boolean mask: access all elements for which the condition is true (the length of the resulting array must be the same as the length of the respective axis)
a<b	less		
a==b	equal		
a!=b	not equal		
a>=b	greater or equal		
a<=b	less or equal		

The syntax follows conventions defined by the Python programming language and the numerics package numpy with few additions. Mathematical functions are mapped on functions provided by the compute engine tensorflow.
