# Supplementary material for: PyRates—A Python framework for rate-based neural simulations
Source: PLoS One. 2019 Dec 16;14(12):e0225900. doi: 10.1371/journal.pone.0225900 (PMC6913930; doi:10.1371/journal.pone.0225900)
Supplement: S2 Table — (PDF) [file pone.0225900.s002.pdf]

Table S2. Overview of preimplemented mathematical functions.

Syntax	Description	Syntax	Description
sin(a)	sine	reshape(a, b)	reorder content of a to shape b (may be a tuple)
cos(a)	cosine	dtype(a)	output data type of a
tan(a)	tangent	squeeze(a)	removes singleton dimensions of array a
abs(a)	absolute value	cast(a, dtype)	Casts array a into new data type dtype
sqrt(a)	square root	randn(a, b, c)	draw c numbers, randomly distributed between a and b
sq(a)	square a^2	ones(a)	initialize an array of shape a filled with ones
exp(a)	exponential e^x	zeros(a)	initialize an array of shape a filled with zeroes
max(a)	maximum	sum(a)	sum over all elements in a
min(a)	minimum	concat(a, b)	concatenate two arrays
argmax(a)	arguments of the maxima	sigmoid(a)	sigmoid function $\frac{1}{1+e^(a)}$
softmax(a)	rescales element of vector a to values between 0 and 1, using the softmax function	range(a, b, c)	create array with integer numbers between a and b of step c
argmin(a)	arguments of the minima	tanh	hyperbolic tangent
round(a)	round to nearest integer	mask(a, b)	apply boolean mask b to array a
roundto(a, b)	round to \textit{b} number of digits		

Mathematical functions are mapped to functions provided by the tensorflow that are named similarly. Additional functions supported by tensorflow may be defined in the frontend. For more information on these functions, please refer to the tensorflow API documentation [20].
